# 11-Hy­droxy-9-(prop-2-en-1-yl)-9,10-dihydro-9,10-propano­anthracen-12-one

**DOI:** 10.1107/S1600536811033538

**Published:** 2011-08-27

**Authors:** Usama Karama, Abdulrahman I. Almansour, Natarajan Arumugam, Ibrahim Abdul Razak, Suhana Arshad

**Affiliations:** a Department of Chemistry, College of Sciences, King Saud University, PO Box 2455, Riyadh 11451, Saudi Arabia; b School of Physics, Universiti Sains Malaysia, 11800 USM, Penang, Malaysia

## Abstract

In the title compound, C_20_H_18_O_2_, the central six-membered ring adopts a boat conformation and the terminal benzene rings make a dihedral angle of 42.66 (4)° with each other. In the crystal structure, the O—H group forms both an intra- and an inter­molecular O—H⋯O hydrogen bond; the former generates an *S*(5) ring and the latter leads to inversion-generated *R*_2_^2^(10) loops. The dimers are further linked into ribbons propagating along the *a* axis by C—H⋯O inter­actions, and the packing is consolidated by weak C—H⋯π inter­actions.

## Related literature

For background to benzocta­mine, see: Wilhelm & Schmidt (1969 ▶); Karama *et al.* (2010*a* ▶). For further synthetic details, see: Karama *et al.* (2010*b* ▶). For ring conformations, see: Cremer & Pople (1975 ▶). For bond-length data, see: Allen *et al.* (1987 ▶). For graph-set descriptors of hydrogen-bond motifs, see: Bernstein *et al.* (1995 ▶).
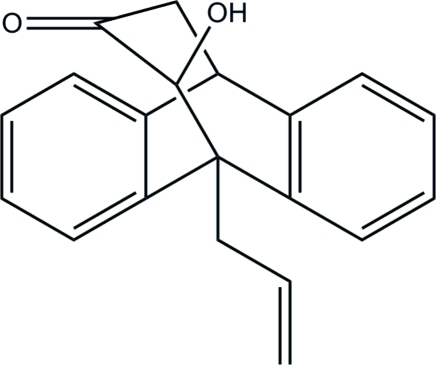


## Experimental

### 

#### Crystal data

C_20_H_18_O_2_
*M* *_r_* = 290.34
Triclinic, 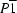

*a* = 7.60940 (1) Å
*b* = 9.16090 (1) Å
*c* = 11.1735 (2) Å
α = 84.202 (1)°
β = 85.707 (1)°
γ = 69.895 (1)°
*V* = 727.02 (2) Å^3^
*Z* = 2
Mo *K*α radiation
μ = 0.08 mm^−1^
*T* = 296 K
0.57 × 0.39 × 0.27 mm

#### Data collection

Bruker SMART APEXII CCD diffractometer
Absorption correction: multi-scan (*SADABS*; Bruker, 2009 ▶) *T*_min_ = 0.954, *T*_max_ = 0.978
14851 measured reflections
5283 independent reflections
4634 reflections with *I* > 2σ(*I*)
*R*_int_ = 0.022

#### Refinement

*R*[*F* ^2^ > 2σ(*F* ^2^)] = 0.045
*wR*(*F* ^2^) = 0.130
*S* = 1.06
5283 reflections
203 parameters
H atoms treated by a mixture of independent and constrained refinement
Δρ_max_ = 0.59 e Å^−3^
Δρ_min_ = −0.26 e Å^−3^

### 

Data collection: *APEX2* (Bruker, 2009 ▶); cell refinement: *SAINT* (Bruker, 2009 ▶); data reduction: *SAINT*; program(s) used to solve structure: *SHELXTL* (Sheldrick, 2008 ▶); program(s) used to refine structure: *SHELXTL*; molecular graphics: *SHELXTL*; software used to prepare material for publication: *SHELXTL* and *PLATON* (Spek, 2009 ▶).

## Supplementary Material

Crystal structure: contains datablock(s) global, I. DOI: 10.1107/S1600536811033538/hb6360sup1.cif

Structure factors: contains datablock(s) I. DOI: 10.1107/S1600536811033538/hb6360Isup2.hkl

Supplementary material file. DOI: 10.1107/S1600536811033538/hb6360Isup3.cml

Additional supplementary materials:  crystallographic information; 3D view; checkCIF report

## Figures and Tables

**Table 1:** Hydrogen-bond geometry (Å, °) *Cg*1 is the centroid of the C1–C6 benzene ring.

*D*—H⋯*A*	*D*—H	H⋯*A*	*D*⋯*A*	*D*—H⋯*A*
O2—H1*O*2⋯O1	0.948 (18)	2.08 (2)	2.600 (2)	113 (2)
O2—H1*O*2⋯O1^i^	0.948 (18)	2.01 (2)	2.8041 (10)	140 (2)
C14—H14*A*⋯O2^ii^	1.00	2.41	3.3909 (12)	166
C17—H17*A*⋯*Cg*1^iii^	1.00	2.92	3.6542 (10)	131
C20—H20*A*⋯*Cg*1^iv^	0.95	2.79	3.6357 (11)	149

Symmetry codes: (i) 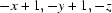; (ii) 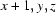; (iii) 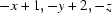; (iv) 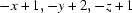.

**Fig. 1.**
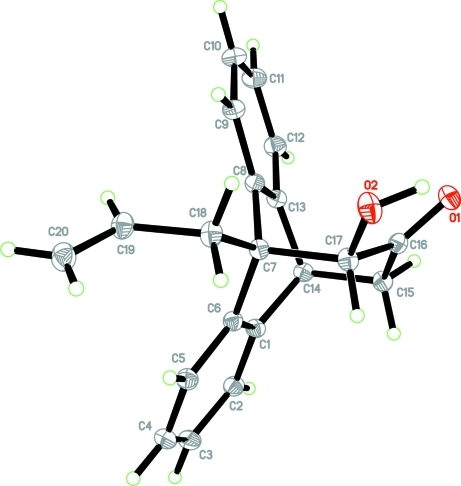
The molecular structure of the title compound, showing 50% probability displacement ellipsoids.

**Fig. 2.**
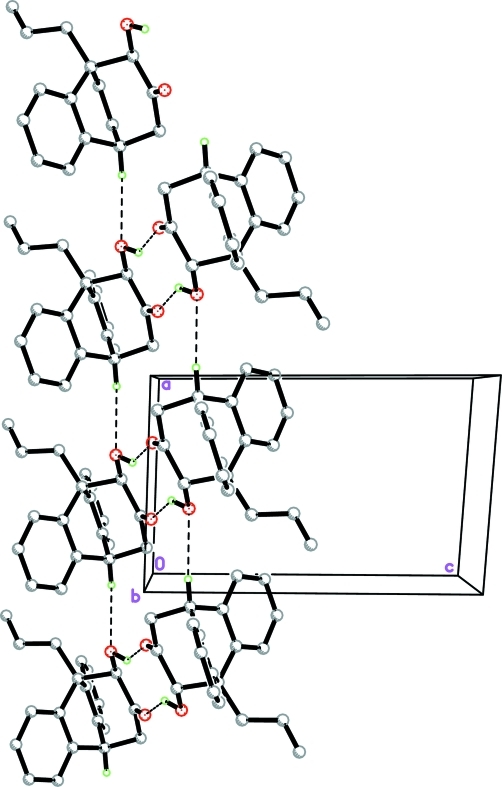
The crystal packing of the title compound. Dashed lines represent the intermolecular hydrogen bonds.

## supplementary crystallographic information

### Comment

Benzoctamine is a clinically useful drug for the treatment of anxiety (Wilhelm & Schmidt, 1969) and our research group recently reported (Karama *et al.*, 2010*a*) the synthesis of bishomobenzoctamine (Karama *et al.*, 2010*b*) as it is a structural mimic of benzoctamine and was derived from anthrone which might be exhibited antidepressant activity. The title compound is the key intermediate for the synthesis of bishomobenzoctamine and its crystal structure is presented here.

In the molecular structure (Fig 1), the central 6-membered ring (C1/C6–C8/C13/C14) adopts a boat conformation with puckering amplitude Q = 0.6082 (9) Å, θ = 91.42 (8)° and φ = 120.69 (9)° (Cremer & Pople, 1975). The terminal benzene rings (C1–C6 and C8–C13) make a dihedral angle of 42.66 (4)° to each other. The bond lengths (Allen *et al.*, 1987) and angles are within normal ranges.

The crystal packing is shown in Fig. 2. The molecules are linked by the intermolecular O2—H1O2···O1 hydrogen bonds (Table 1) and generating *R*^2^_2_(10) ring motifs (Bernstein *et al.*, 1995). These ring motifs are further linked into ribbons along *a* axis *via* intermolecular C14—H14A···O2 hydrogen bonds (Table 1). In addition, the C—H···π interactions (Table 1) which involves C17 and C20 with the phenyl ring (*Cg*1; C1–C6) further stabilized the structure.

### Experimental

To 12-Bromo-9-(prop-2-en-1-yl)-9,10-dihydro-9,10-ethanoanthracen-12-carbaldehyde (Karama *et al.*, 2010*a*) (1 g, 2.85 mmol) in THF (10 ml) was added 1*M* aqueous NaOH (10 ml). The mixture was stirred at room temperature for 4 h, extracted with ether twice, washed with water, dried with MgSO_4_ and the solvent was evaporated *in vacuo* to yield the crude product. The crude product was purified by column chromatography on silica gel (petroleum ether–ethyl acetate 5:1) and product was crystallized from EtOAc to reveal the title compound as colourless blocks.

### Refinement

H1O2 atom attached to the O atom was located from the difference map and refined freely [O—H = 0.948 (19) Å]. The remaining H atoms were positioned geometrically [C—H = 0.95, 0.99 and 1.00 Å] and refined using a riding model with *U*_iso_(H) = 1.2 *U*_eq_(C).

### Crystal data

**Table:**

C_20_H_18_O_2_	*Z* = 2
*M**_r_* = 290.34	*F*(000) = 308
Triclinic, *P*1	*D*_x_ = 1.326 Mg m^−^^3^
Hall symbol: -P 1	Mo *K*α radiation, λ = 0.71073 Å
*a* = 7.60940 (1) Å	Cell parameters from 7628 reflections
*b* = 9.16090 (1) Å	θ = 2.4–32.6°
*c* = 11.1735 (2) Å	µ = 0.08 mm^−^^1^
α = 84.202 (1)°	*T* = 100 K
β = 85.707 (1)°	Block, colourless
γ = 69.895 (1)°	0.57 × 0.39 × 0.27 mm
*V* = 727.02 (2) Å^3^	

### Data collection

**Table:**

Bruker SMART APEXII CCD diffractometer	5283 independent reflections
Radiation source: fine-focus sealed tube	4634 reflections with *I* > 2σ(*I*)
graphite	*R*_int_ = 0.022
φ and ω scans	θ_max_ = 32.7°, θ_min_ = 1.8°
Absorption correction: multi-scan (*SADABS*; Bruker, 2009)	*h* = −11→11
*T*_min_ = 0.954, *T*_max_ = 0.978	*k* = −13→13
14851 measured reflections	*l* = −16→15

### Refinement

**Table:**

Refinement on *F*^2^	Primary atom site location: structure-invariant direct methods
Least-squares matrix: full	Secondary atom site location: difference Fourier map
*R*[*F*^2^ > 2σ(*F*^2^)] = 0.045	Hydrogen site location: inferred from neighbouring sites
*wR*(*F*^2^) = 0.130	H atoms treated by a mixture of independent and constrained refinement
*S* = 1.06	*w* = 1/[σ^2^(*F*_o_^2^) + (0.073*P*)^2^ + 0.1953*P*] where *P* = (*F*_o_^2^ + 2*F*_c_^2^)/3
5283 reflections	(Δ/σ)_max_ < 0.001
203 parameters	Δρ_max_ = 0.59 e Å^−^^3^
0 restraints	Δρ_min_ = −0.26 e Å^−^^3^

### Special details

**Table:**

Experimental. The crystal was placed in the cold stream of an Oxford Cryosystems Cobra open-flow nitrogen cryostat (Cosier & Glazer, 1986) operating at 100.0 (1) K.
Geometry. All e.s.d.'s (except the e.s.d. in the dihedral angle between two l.s. planes) are estimated using the full covariance matrix. The cell e.s.d.'s are taken into account individually in the estimation of e.s.d.'s in distances, angles and torsion angles; correlations between e.s.d.'s in cell parameters are only used when they are defined by crystal symmetry. An approximate (isotropic) treatment of cell e.s.d.'s is used for estimating e.s.d.'s involving l.s. planes.
Refinement. Refinement of *F*^2^ against ALL reflections. The weighted *R*-factor *wR* and goodness of fit *S* are based on *F*^2^, conventional *R*-factors *R* are based on *F*, with *F* set to zero for negative *F*^2^. The threshold expression of *F*^2^ > σ(*F*^2^) is used only for calculating *R*-factors(gt) *etc*. and is not relevant to the choice of reflections for refinement. *R*-factors based on *F*^2^ are statistically about twice as large as those based on *F*, and *R*-factors based on ALL data will be even larger.

### Fractional atomic coordinates and isotropic or equivalent isotropic displacement parameters (Å^2^)

**Table:**

	*x*	*y*	*z*	*U*_iso_*/*U*_eq_	
O1	0.68817 (9)	0.54934 (8)	−0.00032 (6)	0.01745 (14)	
O2	0.37722 (10)	0.68003 (9)	0.12072 (7)	0.01983 (15)	
C1	0.78621 (12)	0.92873 (9)	0.16802 (8)	0.01291 (15)	
C2	0.85293 (13)	1.05379 (10)	0.15089 (8)	0.01569 (16)	
H2A	0.9775	1.0374	0.1203	0.019*	
C3	0.73774 (14)	1.20262 (10)	0.17841 (9)	0.01855 (17)	
H3A	0.7840	1.2873	0.1686	0.022*	
C4	0.55436 (14)	1.22547 (10)	0.22043 (9)	0.01800 (17)	
H4A	0.4745	1.3268	0.2383	0.022*	
C5	0.48627 (12)	1.10130 (10)	0.23671 (8)	0.01547 (16)	
H5A	0.3600	1.1192	0.2643	0.019*	
C6	0.60259 (12)	0.95029 (9)	0.21274 (8)	0.01269 (15)	
C7	0.54044 (11)	0.80676 (9)	0.23205 (8)	0.01258 (15)	
C8	0.70206 (12)	0.66696 (9)	0.28388 (8)	0.01320 (15)	
C9	0.67772 (13)	0.55204 (10)	0.36921 (8)	0.01689 (17)	
H9A	0.5554	0.5601	0.4004	0.020*	
C10	0.83112 (15)	0.42584 (11)	0.40902 (9)	0.02055 (18)	
H10A	0.8126	0.3484	0.4668	0.025*	
C11	1.01105 (14)	0.41270 (11)	0.36456 (9)	0.02113 (19)	
H11A	1.1156	0.3280	0.3934	0.025*	
C12	1.03761 (13)	0.52413 (10)	0.27754 (9)	0.01737 (17)	
H12A	1.1602	0.5145	0.2460	0.021*	
C13	0.88421 (12)	0.64985 (9)	0.23660 (8)	0.01354 (15)	
C14	0.90580 (11)	0.76761 (9)	0.13675 (8)	0.01299 (15)	
H14A	1.0400	0.7611	0.1273	0.016*	
C15	0.84323 (12)	0.73065 (10)	0.01665 (8)	0.01417 (15)	
H15A	0.8081	0.8262	−0.0392	0.017*	
H15B	0.9498	0.6507	−0.0216	0.017*	
C16	0.67958 (12)	0.67252 (10)	0.03665 (8)	0.01311 (15)	
C17	0.50015 (12)	0.76547 (10)	0.10507 (8)	0.01383 (15)	
H17A	0.4382	0.8650	0.0557	0.017*	
C18	0.35765 (12)	0.83894 (10)	0.31087 (8)	0.01568 (16)	
H18A	0.2566	0.9225	0.2683	0.019*	
H18B	0.3227	0.7437	0.3195	0.019*	
C19	0.36715 (13)	0.88655 (11)	0.43476 (8)	0.01713 (17)	
H19A	0.4780	0.8350	0.4775	0.021*	
C20	0.22942 (14)	0.99649 (11)	0.48766 (9)	0.02011 (18)	
H20C	0.1170	1.0500	0.4471	0.024*	
H20A	0.2434	1.0216	0.5660	0.024*	
H1O2	0.412 (3)	0.602 (2)	0.0653 (18)	0.048 (5)*	

### Atomic displacement parameters (Å^2^)

**Table:**

	*U*^11^	*U*^22^	*U*^33^	*U*^12^	*U*^13^	*U*^23^
O1	0.0180 (3)	0.0167 (3)	0.0194 (3)	−0.0076 (2)	0.0007 (2)	−0.0050 (2)
O2	0.0168 (3)	0.0261 (3)	0.0222 (3)	−0.0133 (3)	0.0034 (2)	−0.0089 (3)
C1	0.0129 (3)	0.0129 (3)	0.0139 (4)	−0.0056 (3)	−0.0012 (3)	−0.0005 (3)
C2	0.0174 (4)	0.0158 (3)	0.0161 (4)	−0.0087 (3)	−0.0010 (3)	0.0000 (3)
C3	0.0244 (4)	0.0143 (3)	0.0192 (4)	−0.0094 (3)	−0.0022 (3)	0.0000 (3)
C4	0.0224 (4)	0.0121 (3)	0.0182 (4)	−0.0039 (3)	−0.0025 (3)	−0.0014 (3)
C5	0.0153 (4)	0.0143 (3)	0.0154 (4)	−0.0031 (3)	−0.0016 (3)	−0.0013 (3)
C6	0.0127 (3)	0.0126 (3)	0.0133 (3)	−0.0049 (3)	−0.0010 (3)	−0.0010 (3)
C7	0.0112 (3)	0.0139 (3)	0.0133 (4)	−0.0050 (3)	0.0005 (3)	−0.0020 (3)
C8	0.0141 (3)	0.0131 (3)	0.0132 (4)	−0.0056 (3)	−0.0005 (3)	−0.0010 (3)
C9	0.0201 (4)	0.0159 (4)	0.0157 (4)	−0.0082 (3)	0.0014 (3)	−0.0004 (3)
C10	0.0278 (5)	0.0154 (4)	0.0179 (4)	−0.0078 (3)	−0.0010 (3)	0.0029 (3)
C11	0.0235 (4)	0.0146 (4)	0.0218 (4)	−0.0022 (3)	−0.0042 (3)	0.0017 (3)
C12	0.0151 (4)	0.0157 (4)	0.0193 (4)	−0.0025 (3)	−0.0020 (3)	−0.0010 (3)
C13	0.0134 (3)	0.0128 (3)	0.0145 (4)	−0.0046 (3)	−0.0008 (3)	−0.0009 (3)
C14	0.0115 (3)	0.0134 (3)	0.0147 (4)	−0.0053 (3)	0.0007 (3)	−0.0009 (3)
C15	0.0134 (3)	0.0154 (3)	0.0150 (4)	−0.0069 (3)	0.0025 (3)	−0.0022 (3)
C16	0.0130 (3)	0.0146 (3)	0.0119 (3)	−0.0050 (3)	0.0000 (3)	−0.0010 (3)
C17	0.0116 (3)	0.0162 (3)	0.0151 (4)	−0.0062 (3)	0.0003 (3)	−0.0027 (3)
C18	0.0129 (3)	0.0197 (4)	0.0157 (4)	−0.0071 (3)	0.0017 (3)	−0.0035 (3)
C19	0.0157 (4)	0.0214 (4)	0.0156 (4)	−0.0079 (3)	0.0014 (3)	−0.0032 (3)
C20	0.0201 (4)	0.0223 (4)	0.0188 (4)	−0.0086 (3)	0.0040 (3)	−0.0044 (3)

### Geometric parameters (Å, °)

**Table:**

O1—C16	1.2198 (10)	C10—C11	1.3903 (14)
O2—C17	1.4034 (10)	C10—H10A	0.9500
O2—H1O2	0.948 (19)	C11—C12	1.3929 (13)
C1—C2	1.3957 (11)	C11—H11A	0.9500
C1—C6	1.4023 (11)	C12—C13	1.3955 (12)
C1—C14	1.5039 (11)	C12—H12A	0.9500
C2—C3	1.3945 (13)	C13—C14	1.5122 (12)
C2—H2A	0.9500	C14—C15	1.5622 (12)
C3—C4	1.3901 (14)	C14—H14A	1.0000
C3—H3A	0.9500	C15—C16	1.5081 (12)
C4—C5	1.3946 (12)	C15—H15A	0.9900
C4—H4A	0.9500	C15—H15B	0.9900
C5—C6	1.4024 (12)	C16—C17	1.5329 (12)
C5—H5A	0.9500	C17—H17A	1.0000
C6—C7	1.5342 (11)	C18—C19	1.5069 (13)
C7—C8	1.5369 (12)	C18—H18A	0.9900
C7—C18	1.5441 (12)	C18—H18B	0.9900
C7—C17	1.5822 (12)	C19—C20	1.3281 (13)
C8—C9	1.3988 (12)	C19—H19A	0.9500
C8—C13	1.4081 (12)	C20—H20C	0.9500
C9—C10	1.3939 (13)	C20—H20A	0.9500
C9—H9A	0.9500		
			
C17—O2—H1O2	109.6 (11)	C11—C12—H12A	120.0
C2—C1—C6	120.78 (8)	C13—C12—H12A	120.0
C2—C1—C14	121.59 (8)	C12—C13—C8	120.62 (8)
C6—C1—C14	117.62 (7)	C12—C13—C14	121.59 (8)
C3—C2—C1	120.39 (8)	C8—C13—C14	117.74 (7)
C3—C2—H2A	119.8	C1—C14—C13	109.28 (7)
C1—C2—H2A	119.8	C1—C14—C15	110.01 (7)
C4—C3—C2	119.14 (8)	C13—C14—C15	109.10 (7)
C4—C3—H3A	120.4	C1—C14—H14A	109.5
C2—C3—H3A	120.4	C13—C14—H14A	109.5
C3—C4—C5	120.75 (8)	C15—C14—H14A	109.5
C3—C4—H4A	119.6	C16—C15—C14	112.12 (7)
C5—C4—H4A	119.6	C16—C15—H15A	109.2
C4—C5—C6	120.56 (8)	C14—C15—H15A	109.2
C4—C5—H5A	119.7	C16—C15—H15B	109.2
C6—C5—H5A	119.7	C14—C15—H15B	109.2
C5—C6—C1	118.33 (7)	H15A—C15—H15B	107.9
C5—C6—C7	123.79 (7)	O1—C16—C15	120.54 (8)
C1—C6—C7	117.88 (7)	O1—C16—C17	118.56 (8)
C6—C7—C8	109.17 (7)	C15—C16—C17	120.90 (7)
C6—C7—C18	111.72 (7)	O2—C17—C16	109.59 (7)
C8—C7—C18	112.73 (7)	O2—C17—C7	109.46 (7)
C6—C7—C17	108.29 (7)	C16—C17—C7	112.40 (7)
C8—C7—C17	107.27 (6)	O2—C17—H17A	108.4
C18—C7—C17	107.46 (7)	C16—C17—H17A	108.4
C9—C8—C13	118.50 (8)	C7—C17—H17A	108.4
C9—C8—C7	124.06 (8)	C19—C18—C7	115.09 (7)
C13—C8—C7	117.33 (7)	C19—C18—H18A	108.5
C10—C9—C8	120.67 (8)	C7—C18—H18A	108.5
C10—C9—H9A	119.7	C19—C18—H18B	108.5
C8—C9—H9A	119.7	C7—C18—H18B	108.5
C11—C10—C9	120.33 (8)	H18A—C18—H18B	107.5
C11—C10—H10A	119.8	C20—C19—C18	123.72 (9)
C9—C10—H10A	119.8	C20—C19—H19A	118.1
C10—C11—C12	119.80 (8)	C18—C19—H19A	118.1
C10—C11—H11A	120.1	C19—C20—H20C	120.0
C12—C11—H11A	120.1	C19—C20—H20A	120.0
C11—C12—C13	120.02 (9)	H20C—C20—H20A	120.0
			
C6—C1—C2—C3	0.18 (13)	C9—C8—C13—C12	−2.45 (13)
C14—C1—C2—C3	178.71 (8)	C7—C8—C13—C12	−178.77 (8)
C1—C2—C3—C4	−1.48 (14)	C9—C8—C13—C14	174.81 (8)
C2—C3—C4—C5	0.90 (14)	C7—C8—C13—C14	−1.50 (11)
C3—C4—C5—C6	1.00 (14)	C2—C1—C14—C13	136.26 (8)
C4—C5—C6—C1	−2.27 (13)	C6—C1—C14—C13	−45.17 (10)
C4—C5—C6—C7	178.01 (8)	C2—C1—C14—C15	−103.98 (9)
C2—C1—C6—C5	1.69 (13)	C6—C1—C14—C15	74.60 (9)
C14—C1—C6—C5	−176.90 (8)	C12—C13—C14—C1	−138.29 (8)
C2—C1—C6—C7	−178.58 (8)	C8—C13—C14—C1	44.47 (10)
C14—C1—C6—C7	2.83 (11)	C12—C13—C14—C15	101.38 (9)
C5—C6—C7—C8	−140.45 (8)	C8—C13—C14—C15	−75.85 (9)
C1—C6—C7—C8	39.84 (10)	C1—C14—C15—C16	−83.63 (8)
C5—C6—C7—C18	−15.07 (11)	C13—C14—C15—C16	36.24 (9)
C1—C6—C7—C18	165.21 (8)	C14—C15—C16—O1	−125.97 (9)
C5—C6—C7—C17	103.08 (9)	C14—C15—C16—C17	53.24 (10)
C1—C6—C7—C17	−76.64 (9)	O1—C16—C17—O2	4.54 (11)
C6—C7—C8—C9	143.59 (8)	C15—C16—C17—O2	−174.69 (7)
C18—C7—C8—C9	18.81 (12)	O1—C16—C17—C7	126.48 (8)
C17—C7—C8—C9	−99.29 (9)	C15—C16—C17—C7	−52.74 (10)
C6—C7—C8—C13	−40.32 (10)	C6—C7—C17—O2	−156.87 (7)
C18—C7—C8—C13	−165.10 (7)	C8—C7—C17—O2	85.43 (8)
C17—C7—C8—C13	76.81 (9)	C18—C7—C17—O2	−36.03 (9)
C13—C8—C9—C10	1.83 (13)	C6—C7—C17—C16	81.11 (8)
C7—C8—C9—C10	177.88 (8)	C8—C7—C17—C16	−36.59 (9)
C8—C9—C10—C11	0.19 (15)	C18—C7—C17—C16	−158.05 (7)
C9—C10—C11—C12	−1.64 (15)	C6—C7—C18—C19	−59.15 (10)
C10—C11—C12—C13	1.02 (15)	C8—C7—C18—C19	64.23 (10)
C11—C12—C13—C8	1.05 (14)	C17—C7—C18—C19	−177.80 (7)
C11—C12—C13—C14	−176.11 (8)	C7—C18—C19—C20	139.88 (9)

### Hydrogen-bond geometry (Å, °)

**Table:**

Cg1 is the centroid of the C1–C6 benzene ring.

**Table:**

*D*—H···*A*	*D*—H	H···*A*	*D*···*A*	*D*—H···*A*
O2—H1O2···O1	0.948 (18)	2.08 (2)	2.600 (2)	113 (2)
O2—H1O2···O1^i^	0.948 (18)	2.01 (2)	2.8041 (10)	140 (2)
C14—H14A···O2^ii^	1.00	2.41	3.3909 (12)	166
C17—H17A···Cg1^iii^	1.00	2.92	3.6542 (10)	131
C20—H20A···Cg1^iv^	0.95	2.79	3.6357 (11)	149

Symmetry codes: (i) −*x*+1, −*y*+1, −*z*; (ii) *x*+1, *y*, *z*; (iii) −*x*+1, −*y*+2, −*z*; (iv) −*x*+1, −*y*+2, −*z*+1.
